# Individually Tailored Physiotherapy in Persons With Respiratory Symptoms Related to Post‐Acute Sequelae of COVID‐19: A Feasibility Study With Mixed Methods

**DOI:** 10.1002/hsr2.71367

**Published:** 2025-10-21

**Authors:** Marcus Lo, Lauren Eiriksson, Simone Hunter, Rosie Twomey, Kate Skolnik, Joel Chen, Elnaz Ehteshami Afshar, Jason Weatherald, Rachel K. Lim

**Affiliations:** ^1^ Department of Medicine, Cumming School of Medicine University of Calgary Calgary Alberta Canada; ^2^ Faculty of Kinesiology University of Calgary Calgary Alberta Canada; ^3^ Breathe Well Physio Calgary Alberta Canada; ^4^ Department of Medicine, Cumming School of Medicine, Division of Respiratory Medicine University of Calgary Calgary Alberta Canada; ^5^ Department of Community Health Sciences, Cumming School of Medicine University of Calgary Calgary Alberta Canada

**Keywords:** long‐covid, quality of life, rehabilitation

## Abstract

**Background and Aims:**

Post‐acute sequelae of COVID‐19 (PASC) commonly present with persistent respiratory symptoms, even in individuals with normal chest imaging and pulmonary function. Given the heterogeneity within this population, a personalized approach to respiratory physiotherapy could improve outcomes. The purpose of this study was to assess the feasibility and impact of a tailored respiratory physiotherapy program on health‐related quality of life (QoL), functional impairment, and patient‐reported outcome measures (PROMs) in individuals with persistent respiratory symptoms due to PASC.

**Methods:**

A single‐arm, open‐label trial was conducted with 13 adults diagnosed with PASC, recruited from Long COVID clinics in Calgary, Canada. Participants underwent an 8‐session personalized physiotherapy program, including education, breathing exercises, and strengthening. Feasibility was measured through recruitment, retention, and session completion rates. PROMs were collected at baseline and post‐intervention, and qualitative interviews explored participant perspectives.

**Results:**

The program was highly feasible, with 100% retention and a 99% completion rate. Significant improvements were observed in QoL, functional status (Post COVID‐19 Function Status scale), and self‐efficacy scores. The 6‐min walk test showed clinically meaningful improvements in three out of seven participants. Qualitative interviews (*n* = 8) identified three main themes: struggles with PASC, positive aspects of the program, and benefits from completing it. Participants valued the personalized approach, heart rate monitors, flexible scheduling, and a hybrid of in‐person and virtual sessions, reporting increased confidence, improved symptom management, and better mental health.

**Conclusion:**

A personalized respiratory physiotherapy program is feasible and may benefit individuals with PASC. Larger trials are needed to assess long‐term efficacy and scalability.

**Trial Registration:**

ClinicalTrials.gov identifier: NCT05040893

## 1 Introduction

The global coronavirus disease 2019 (COVID‐19) pandemic has resulted in catastrophic loss of life and significant morbidity among survivors. Those who have persistent symptoms for greater than 12 weeks postinfection that are not otherwise explained are considered to have “Long‐Covid” or post‐acute sequelae of COVID‐19 (PASC) [1, 2]. PASC is estimated to affect at least 10% of infected people, which necessitates effective and accessible interventions to manage this condition [2]. Various pathophysiologic mechanisms have been proposed, and the heterogeneity of this condition makes it difficult to diagnose and manage [2].

Fatigue and respiratory complaints, including dyspnea, cough, and poor exertional tolerance, are among the most frequently reported symptoms [3]. Many COVID‐19 survivors will have abnormalities on pulmonary function testing (PFT) (e.g., reduced diffusion capacity) and chest imaging (e.g., ground glass opacities), with some even having more serious disease, such as pulmonary fibrosis or pulmonary vascular disease [4, 5]. Yet, a large proportion of individuals with respiratory symptoms have no objective impairment on PFTs or chest imaging [6, 7]. In this population, potential causes of respiratory symptoms can include breathing pattern disorder (BPD), diaphragmatic weakness, post‐exertional malaise (PEM), postural orthostatic tachycardia syndrome, and general deconditioning [3, 8]. There are currently no approved treatments for PASC despite the significant impact on quality of life and function [9, 10]. International guidelines recommend multidisciplinary approaches to recovery from PASC, but rehabilitation access and programming vary widely across centers [9]. Guidelines also emphasize the role of respiratory physiotherapy in the recovery of ambulatory individuals with PASC, focusing on education, breathing retraining strategies, pacing, and modest goal setting [11, 12, 13].

Physiotherapy benefits are recognized in hospitalized patients; however, tailored physiotherapy may also play an instrumental role in the recovery of ambulatory patients with PASC without overt cardiorespiratory disease and normal investigations. Multiple studies have demonstrated the positive impact of physiotherapy programs on PASC symptoms, although many implemented aerobic and resistance exercises while lacking respiratory‐focused interventions like breathing pattern retraining [14, 15, 16, 17]. Patient perspectives from engaging in physiotherapy are also underexplored in the literature. Given the complexity and heterogeneity of people suffering from respiratory symptoms related to PASC, the authors hypothesized that a tailored approach to respiratory physiotherapy interventions may be effective in alleviating symptoms and improving health‐related quality of life.

The authors conducted a pragmatic open‐label trial to determine the feasibility and impact of an individualized respiratory physiotherapy program on health‐related quality of life and other patient‐reported outcome measures (PROMs) among individuals with persistent respiratory symptoms related to PASC. As part of the study, a qualitative analysis was also done to explore participant perspectives on the perceived benefits and barriers to completing the tailored respiratory physiotherapy program delivered by a specialized physiotherapist.

## 2 Methods

### 2.1 Study Design

This was a single‐arm, open‐label trial investigating the feasibility of a tailored, weekly pulmonary physiotherapy program delivered individually by a specialized physiotherapist. The study was registered at clinicaltrials.gov (identifier NCT05040893) and approved by the University of Calgary ethics board (REB21‐0767). All participants included in the analysis provided written informed consent for study participation.

### 2.2 Study Setting and Participants

Between March 2022 and March 2023, adults aged 18 years or older were recruited from two specialized respiratory PASC clinics in Calgary, Canada. No formal sample size calculation was performed due to the nature of this being a pilot study, and the sample size was determined based on recommendations that a sample size of at least 12 provides good feasibility and precision in pilot studies [18]. Participants had to have persistent respiratory symptoms, have been diagnosed with PASC by a physician, and have a previous positive polymerase chain reaction (PCR) test for SARS‐CoV‐2 to be eligible. Exclusion criteria included the presence of abnormal PFTs (according to recent guidelines) [19], current participation in any other physiotherapy/rehabilitation program, pregnancy, or known history of the following conditions: chronic lung disease, cardiovascular disease, venous thromboembolism within the past year, or myalgic encephalomyelitis/chronic fatigue syndrome.

Following consent, participant demographics, details of their COVID‐19 illness, and current symptoms of PASC were collected at enrollment. Baseline measurements included their most recent PFTs, dyspnea score (modified Medical Research Council [mMRC]), and Post COVID‐19 Function Status (PCFS) [20]. Participants completed the brief DePaul Symptom Questionnaire‐PEM questionnaire to screen for PEM [21]. Participants also completed the EQ‐5D‐VAS, cough visual analog score (VAS), and PROMIS measures of self‐efficacy (for managing daily activities and managing symptoms) before their initial physiotherapy session and after their last session [22, 23]. Participants also gave a Patient Global Impact of Change (PGIC) score after the last session [24]. Participants who screened negative for PEM completed a 6‐min walk test (6MWT) before and after the treatment program; if PEM was present, participants were given the option of performing a 1‐min sit‐to‐stand test (STST) instead [25, 26].

### 2.3 Study Intervention

A registered physiotherapist with expertise in cardiopulmonary rehabilitation and training in BPD created an individualized treatment plan that was delivered in person and virtually over several weeks (8 sessions total, each lasting 1 h). Participants were allowed a maximum of one session per week but could schedule them further apart based on their schedule and preference. The first session was done in person, but subsequent sessions could be done virtually based on participant preference. Participants were provided with activity trackers to help guide home exercises and activities between sessions. The design of the program considered participant goals, current and past physical fitness, symptom stability, orthostatic tolerance, current physical/cognitive limitations, and equipment availability.

The physiotherapy intervention focused on three main pillars for intervention: (1) education and self‐management strategies, including the Stop‐Rest‐Pace approach [27] and the use of wearable activity and real‐time heart rate monitors to safely guide pacing and activity, (2) Breathing pattern education and retraining (including diaphragmatic, relaxed, and paced breathing exercises), and (3) Return‐to‐activity exercise programming (e.g., postural, aerobic, and whole‐body strengthening exercises). Therapy emphasized symptom management strategies with a focus on improving the participant's functional ability to perform everyday tasks.

### 2.4 Quantitative Analysis

The primary outcome was the feasibility of the intervention, defined as a combined outcome including: (1) recruitment of at least 12 participants during the study (*N* based on pilot study design [18]); (2) retention of ≥ 70% of participants; and (3) completion of ≥ 70% of all supervised physiotherapy sessions. Secondary outcomes included changes in PROMs, which assessed health‐related quality of life, level of functional impairment, and self‐efficacy for symptom management. Pre‐ and post‐differences in scores were analyzed using either paired *t*‐tests or Wilcoxon signed rank tests, depending on data distribution. Statistical significance was assumed at *p*‐value < 0.05. Effect sizes were determined using Cohen's *d*, with classification as small (*d* = 0.2 or below), moderate (*d* = 0.5–0.79), large (*d* = 0.8–1.29), or very large (d = 1.3 or higher) [28]. All analyses were performed using STATA 16.1 (StataCorp LP, College Station, USA).

### 2.5 Qualitative Analysis

Upon completion of the sessions, participants underwent a semi‐structured phone interview lasting approximately 30 min by a trained researcher who was not involved with either the participant's routine clinical care or the study intervention. The interview followed a standardized interview guide (see Appendix S1), which contained a series of open‐ended questions and probes to encourage participants to provide more detail, elaborate, or clarify what they were saying. The interview guide was informed by the Theoretical Framework of Acceptability [29]. All interviews were audio‐recorded, transcribed verbatim for data analysis, and anonymized to protect participant confidentiality. Transcripts were imported and analyzed using NVivo 12 software. Transcripts were analyzed using a hybrid inductive‐deductive approach using reflexive thematic analysis, where two authors (M.L. and R.K.L.) reviewed the transcripts, familiarized themselves with the data, and coded the transcripts [30]. Following this, codes were sorted into potential themes and subthemes through an iterative process, thus allowing the collective participant voice to emerge. Discrepancies in coding were discussed to see if consensus could be reached, but a third member was consulted if needed.

## 3 Results

### 3.1 Participant Population

**Figure 1:**
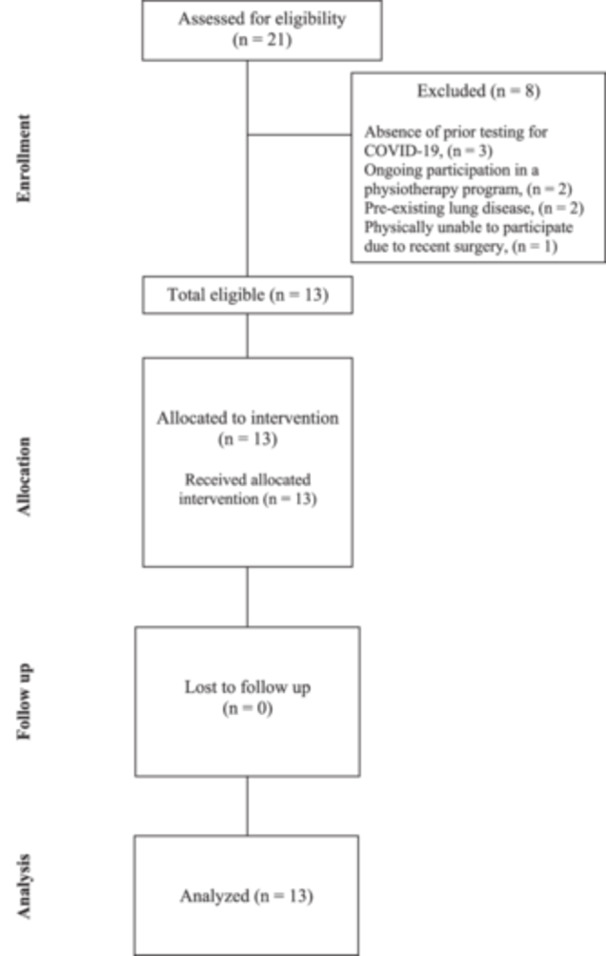
Flow diagram of participant flow through the enrollment, allocation, follow‐up, and analysis phases of the study.

A total of 21 individuals were screened, and 13 were eligible and enrolled. Reasons for exclusion included absence of prior testing for COVID‐19 (*n* = 3), ongoing participation in a physiotherapy program (*n* = 2), existing significant lung disease (*n* = 2), and an inability to physically participate due to surgery (*n* = 1) (see Figure 1). Of 13 participants, 11 were female, the mean age was 45 years (± 14 years), and the mean BMI was 25.5 (± 3.7 kg/m^2^). The median time since COVID‐19 infection was 38 months (interquartile range [IQR]: 19–53), and only one participant had been hospitalized at the time of infection (non‐ICU). The median PCFS was 2 (IQR: 2, 2.5). The most common symptoms were dyspnea and fatigue (both 85%), followed by impaired exertional tolerance (53%), chest pain (38%), and cough (30%). Comorbidities were infrequent, and most were non‐smokers (92%). Baseline characteristics are presented in Table 1.

**Table 1:** Demographics.

Baseline characteristics (*n* = 13)	
Sex, *n* (%)	
Female	11 (85%)
Male	2 (15%)
Mean age at enrollment (SD)	45.8 (14)
Median time since COVID‐19 diagnosis to enrollment (IQR)	38 (19–53)
Hospitalized, *n* (%)	
Yes	1 (8%)
No	12 (92%)
Symptoms, *n* (%)	
Dyspnea	11 (85%)
Fatigue	11 (85%)
Cough	4 (31%)
Chest pain	5 (38%)
Lower exertion	7 (54%)
Dizziness	2 (15%)
Brain fog	3 (23%)
Depressed mood	1 (7%)
Employed or student, *n* (%)	9 (69%)
Mean weight in kg (SD)	71.3 (12.4)
Mean BMI (kg/cm^2^) (SD)	25.5 (3.7)
Smoker, *n* (%)	1 (8%)
Mean FEV1 % predicted (SD)	104% (15)
Mean FVC % predicted (SD)	106% (15)
Mean DLCO % predicted (SD)	106% (12)
Baseline mMRC, *n* (%)	
0	1 (8%)
1	6 (46%)
2	4 (31%)
3	1 (8%)
4	1 (8%)
Mean 6MWD in meters* (SD)	447 (70)
Comorbidities, *n* (%)	
Depression	1 (7%)
Fibromyalgia	1 (7%)
Asthma (controlled)	2 (15%)
Anxiety	1 (7%)
Osteoarthritis	1 (7%)
Malignancy	1 (7%)

Abbreviations: 6MWD, 6‐min walk distance; BMI, body mass index; DLCO, diffusing capacity for carbon monoxide; FEV1, forced expiratory volume in 1 s; FVC, forced vital capacity; IQR, interquartile range; mMRC, modified Medical Research Council Dyspnea Scale; SD, standard deviation.

* Data available for 9 of 13 participants.

### 3.2 Feasibility and Details of Intervention

The components of each participant's physiotherapy program are detailed in Table 2. The intervention was considered feasible based on (1) the recruitment of 13 participants, (2) 100% retention, and (3) completion of 99% of supervised sessions. All participants completed post‐intervention assessments, but two participants declined the post‐6MWT. During the trial, participants were given the flexibility to schedule their sessions at their convenience. Therefore, the range of duration spanned 13–31 weeks (average 20 weeks). No adverse events occurred during the sessions.

**Table 2:** Program details.

Participant	Number of sessions	Sessions in clinic/virtual	Interventions during all sessions
1	8	4/4	▪ Pacing and activity planning ▪ Breathing pattern education and retraining ▪ Strengthening exercises ▪ Restorative movement ▪ Other (return to work consideration and planning)
2	7	2/5	▪ Pacing and activity planning ▪ Breathing pattern education and retraining ▪ Restorative movement ▪ Other (emotional stress management strategies)
3	8	5/3	▪ Pacing and activity planning ▪ Breathing pattern education and retraining ▪ Airway clearance techniques ▪ Inspiratory muscle training
4	8	3/5	▪ Pacing and activity planning ▪ Breathing pattern education and retraining ▪ Strengthening exercises
5	8	5/3	▪ Pacing and activity planning ▪ Breathing pattern education and retraining ▪ Cough control strategies ▪ Cardiorespiratory exercise
6	8	6/2	▪ Pacing and activity planning ▪ Breathing pattern education and retraining ▪ Restorative movement
7	8	6/2	▪ Pacing and activity planning ▪ Breathing pattern education and retraining ▪ Cough control strategies ▪ Cardiorespiratory exercises ▪ Strengthening exercises
8	8	6/2	▪ Pacing and activity planning ▪ Breathing pattern education and retraining ▪ Cardiorespiratory exercise ▪ Strengthening exercises
9	8	8/0	▪ Pacing and activity planning ▪ Breathing pattern education and retraining ▪ Cough control strategies ▪ Cardiorespiratory exercise ▪ Strengthening exercises
10	8	8/0	▪ Pacing and activity planning ▪ Breathing pattern education and retraining ▪ Strengthening exercises
11	8	8/0	▪ Pacing and activity planning ▪ Breathing pattern education and retraining ▪ Strengthening exercises ▪ Cardiorespiratory exercises
12	8	7/1	▪ Pacing and activity planning ▪ Breathing pattern education and retraining ▪ Strengthening exercise ▪ Cardiorespiratory exercise
13	8	8/0	▪ Pacing and activity planning ▪ Breathing pattern education and retraining ▪ Cough control strategies ▪ Strengthening exercises ▪ Inspiratory muscle training ▪ Cardiorespiratory exercise

### 3.3 Effectiveness of the Intervention

Table 3 outlines the statistical comparison between baseline and post‐intervention results. Overall, there were significant improvements in measures of health‐related quality of life (EQ‐5D index and EQ‐VAS), as well as in individual EQ‐5D domains of usual activities, pain/discomfort, and anxiety/depression. Level of functioning (PCFS) and cough severity (VAS) were also improved. Participants' self‐efficacy for managing symptoms improved across all domains. For self‐efficacy to manage daily activities, only confidence to perform household chores improved (*p* = 0.0061). All participants perceived improvements following the program, with the majority (10 of 13) feeling “much” to “very much improved” on the PGIC test. Seven participants completed the 6MWT at both baseline and post‐intervention, and changes were not statistically significant. Increases in 6MWT distance were clinically meaningful (using an MCID of 30 m) in 3/7 participants [31]. Four participants did 1‐min STST instead of 6MWT; all had an improvement in repetitions (range: 4–20).

**Table 3:** Comparison of secondary outcome measures at baseline versus post‐intervention (*n* = 13).

Secondary outcome	Baseline	Post‐intervention	*p*‐value	Cohen's *d*
EQ‐5D index	0.65 (0.23)	0.81 (0.14)	**0.004**	−0.79
EQ mobility	1.92 (0.76)	1.62 (0.77)	0.3033	0.4
EQ self‐care	1.31 (0.48)	1.38 (0.51)	0.5845	−0.16
EQ usual activities	2.08 (0.76)	1.08 (0.64)	**0.0003**	1.42
EQ pain/discomfort	2.77 (1.24)	2 (0.82)	**0.0112**	0.73
EQ anxiety/depression	2.62 (1.12)	2.23 (0.93)	0.0544	0.37
EQ‐VAS	60 (17)	72 (17)	**0.0003**	−0.71
Cough VAS	21 (22)	11 (11)	0.06	0.6
PCFS	2.08 (0.64)	1.54 (0.88)	**0.0149**	0.7
Self ADL, household chores	2.85 (1.14)	3.54 (1.05)	**0.0061**	−0.63
Self ADL, shopping/errands	3.15 (1.07)	3.46 (1.13)	0.3033	−0.28
Self ADL, ambulating in house	4.38 (0.96)	4.31 (1.03)	0.9375	0.08
Self ADL, regular exercise	2.69 (0.85)	3 (1.22)	0.3925	−0.29
Self symptoms, managing during usual activities	2.69 (1.03)	3.69 (1.03)	**0.0020**	−0.97
Self symptoms, interfering with friends/family	2.75 (1.06)	3.58 (0.99)	**0.0105**	−0.78
Self symptoms, managing in public	3.08 (0.64)	3.69 (0.85)	**0.0136**	−0.81
Self symptoms, managing with doctor	3 (0.71)	3.77 (1.01)	**0.0024**	−0.88
6MWT distance (m)*	476 (46)	506 (47)	0.2094	
STS**	26 (12)	37 (12)	0.0669	
PGIC	—	1.85 (0.8)	—	

*Note:* Data are mean (standard deviation). Comparisons were done using paired *t*‐test or Wilcoxon signed‐rank test and effect sizes by Cohen's *d*. EQ‐5D index, EuroQol Group Association five‐domain index. Index has an upper boundary of 1 that indicates full health, whereas 0 represents death. EQ‐VAS scores range from 0, being the worst health imaginable, to 100, being the best health imaginable. Cough VAS scores range from 0, meaning no cough present, to 100, meaning the worst cough imaginable. PCFS scores range from 0 to 4, with a higher score indicating a more severe limitation in everyday life. Self ADL scores range from 1, meaning “I am not at all confident,” to 5, indicating “I am very confident.” Self symptoms scores range from 1, meaning “I am not at all confident,” to 5, indicating “I am very confident.” PGIC scores range from 1 (very much improved) to 7 (very much worse). Bold values are statistical significance (*p*‐value < 0.05).

Abbreviations: 6MWT, 6‐min walk test; Cough VAS, cough visual analog scale; EQ‐VAS, EuroQol visual analog scale; PCFS, post‐COVID function status; PGIC, patient global impression of change; Self ADL, self‐efficacy for managing various activities of daily living self symptoms, self‐efficacy for managing symptoms in various settings; STS, sit‐to‐stand in 1 min.

* Results available for 7/13 participants.

** Results available for 4 participants who did STS in lieu of 6MWT.

### 3.4 Qualitative Results

Eight participants underwent an interview after the intervention, while the remainder either declined or could not be reached. Based on these 8 individual interviews, 3 themes with 11 subthemes emerged (see Table 4): (1) struggles from living with PASC; (2) positive aspects of the program; and (3) benefits of completing the program. The response from participants was overwhelmingly positive, with several stating that this program would be beneficial for others and should be accessible.

**Table 4:** Qualitative analysis of one‐on‐one interviews.

Themes	Subthemes	Statement example
Struggles from living with PASC	Diagnostic uncertainty	“After that I had debilitating symptoms linger on for quite some time after that. And then just going to doctors. Nobody was able to kind of help. They don't, they didn't know what was going on.” – Participant 1 “Nobody can tell you what's going on, and you rely on your medical providers like so much.” – Participant 1 “I had seen multiple doctors, been in and out of emerg, like you name it, and everyone kind of shrugs their shoulders and says we don't know what to do with you. Go home, here's some more pain medication. Just stay home until you feel better, and you can tell me when that would be.” – Participant 6
	Difficulty managing symptoms	“I've just been really struggling with like, energy levels and pain and it's been difficult to manage.” – Participant 10 “I just didn't know how to deal with my symptoms, I didn't know, I didn't know how to make them get better.” – Participant 5 “I felt very discouraged with my symptoms, and how they were affecting my life, and I didn't know how to resolve them.” – Participant 5
	Frustration	“I was able to do this like last year, and now I can't do it, or like I run up the stairs and be out of breath like, that's stupid. Like I should be able to do that.” – Participant 6 “keeping me from doing things that I would normally do on my daily life. Like going into stores, or going for a bike ride or going for a hike with friends, and not being able to do that, those were the other distressing things.” – Participant 9
	Hopelessness	“When they tell you they don't know and you're like, well, what am I supposed to do now?” – Participant 1 “I was quite emotional because I had experienced crashes and setbacks in terms of my well‐being. And yeah, I, I mean I left feeling emotional because I was expressing so much emotion and feeling quite stuck and frustrated.” – Participant 7
Positive aspects of the program	Personalized treatment	“[The physiotherapist] treated me according to what I needed versus just, you know, having a formula.” – Participant 7 “…we always talked about what happened in those two weeks prior in between this last session, and anything that I wasn't happy with which might need changing. Or if I was happy with them or if I needed, if I wanted to do more, or have more breathing exercises in.” – Participant 12
	Physical touch	“It was definitely a lot more hands on, so it was easier to like, learn the stretches properly and the movement properly.” – Participant 11 “I actually found like the hands‐on stuff was actually really good. So like she would always say like, can I touch your shoulder, or can I touch your belly, or whatever. But that was actually, I actually found the most value from when she, physically like, put her hands on me, showed me you know what, what muscle I should be using, or how I should change my posture, or whatever, so like, to, to me that was actually maybe some of the most valuable things, is when she was physically like touching me.” – Participant 5 “…and then also the quality of touch. When she was trying to help me with my breathing patterns. So, you know, like being able to actually put her hand on me and, and measure my breath, and also being able to observe how I was breathing.” – Participant 7
	Activity tracker/monitor (Garmin watch)	“It gives me like, when do I need to slow down and like so awareness of pacing and so that was that was really handy you know.” – Participant 11 “Like even for myself, I found that, might be like, walking like I initially I wouldn't feel the pain in my chest or the out of breath. But my, the heart monitor would say abnormal heart rate, take a break. So that I found really helped. Whereas before if I didn't have that, I would have continued my walk, or do whatever task I was doing and then when I would be done…” – Participant 1 “I like when I go for a walk or doing any activity, and then the heart rate monitor would say abnormal heart rate, that would really kind of trigger, trigger and tell me that okay, you need to take a break and take a pause and don't rush kind of thing to make things worse. So yeah, that helped.” – Participant 1
	Flexible scheduling	“Spacing the sessions out allowed time for like the base information on the first couple of sessions close together was very beneficial. But then spacing them out later you know, allowed me time to heal and benefit from them.” – Participant 8 “October to February, to have my full 8 sessions, and I thought they were good. It gave me the opportunity to end more time to actually focus on the previousing techniques that she was teaching me. And so I, I feel like every time that I went to a session, or had a session I had more improvement at each one, because there was a length of time between.” – Participant 9
	Hybrid model of delivery	“The Zoom sessions were like they're kind of convenient right, cause you don't have to drive anywhere…” – Participant 5 “she could see, just by looking at your body language. So I think actually in person is more important than virtual really, because then she can actually assess you as you're doing things.” – Participant 12
Benefits of completing the program	Confidence	“My confidence at the end of the study is dramatically more than it was before.” – Participant 5 “I gained a lot of confidence in my ability to yeah, deal with how I'm doing.” – Participant 8 “There is a large time when I was, you know, basically scared to do anything because I didn't know how it was gonna work, and how I was gonna react. Now I feel like I can plan with some effectiveness, doing the things that I wanna do or need to do, and have, have a path forward.” – Participant 8
	Management strategies	“You're confident for managing your symptoms. Like the how did the, what I learned like. Or like the pacing.” – Participant 11 “I think doing the breathing exercises and all that I did learn that it was manageable. So yeah, and even the fatigue, learning how to pace myself and not overexert. And now how easy it so I just kind of manage it sometimes and I'm, I guess I'm fortunate right now that I'm on a leave for work so I'm able to kind of do that.” – Participant 1 “…at the end of the study I still do have the symptoms, but I know how to resolve them…” – Participant 5
	Mental health benefit	“my mental health is in a much better place as well.” – Participant 1 “It improved my outlook, and pretty much changed things for the positive for me.” – Participant 7 “It impacted me in a very positive way. I come out of it like, in a way better position than I did going in.” – Participant 9 “(The physiotherapist) was a little bit of physiotherapist slash therapist for me to be honest…” – Participant 1

#### 3.4.1 Struggles From Living With PASC

Subthemes found were: (1) diagnostic uncertainty, (2) difficulty managing symptoms, (3) frustration, and (4) hopelessness. We found that subthemes (1) and (4) were intricately related to each other. Many participants experienced diagnostic uncertainty regarding their PASC symptoms as they presented with chronic, debilitating symptoms in the context of normal cardiopulmonary testing. This highlighted a dilemma that was twofold – both on the part of the clinician attempting to make a unifying diagnosis in the face of normal objective testing, and on the part of the participant not receiving a diagnosis to explain their myriad of symptoms. A lack of abnormalities in standard testing creates an additional barrier for participants seeking care, because these test results are often used as entry criteria for rehabilitation programs.

We also found that subthemes (2) and (3) were closely related. Given that many participants were unable to access the specialized care they needed, they continued to struggle with chronic symptoms that impacted their daily functioning and health‐related quality of life. An inability to perform everyday tasks at their previous level of functioning led to frustration, which also negatively impacted their mental health. This vicious cycle, evident in several interviews, led to a sense of hopelessness with present circumstances.

#### 3.4.2 Positive Aspects of the Program

Five subthemes captured participants' perspectives of the physiotherapy program, mainly highlighting the strengths of the intervention. The subthemes were: (1) personalized treatment, (2) physical touch, (3) use of an activity tracker/monitor (Garmin), (4) flexible scheduling, and (5) hybrid model of delivery.

The benefit of having personalized treatment was expressed by multiple participants. Having physiotherapy that was tailored to participants' individual needs allowed participants to focus their efforts in areas that were causing their specific physical symptoms (dysfunctional breathing, issues with pacing, etc.). Another subtheme was the aspect of physical touch, which many participants found beneficial when comparing the in‐person sessions compared to the virtual sessions. Many participants found that the quality of physical touch delivered by a physiotherapist not only helped them understand how to better perform certain exercises, but also localized many areas that were implicated in patterns of dysfunctional breathing or posture that may have been exacerbating their symptoms.

Many participants also found the use of an activity tracker/monitor (Garmin watch) to be helpful in making progress. More specifically, participants found that having an activity monitor that provided real‐time feedback about their heart rate allowed them to pace themselves more effectively. The monitors also provided objective correlates to participants' symptoms, which they did not previously have available. This in turn allowed them to adjust their activity levels to avoid symptom exacerbation, which allowed many participants to increase their perceived endurance and activity tolerance.

The scheduling of the weekly sessions was flexible based on participant availability. Many participants stated that this allowed them to complete all the sessions at their desired pace. The hybrid of in‐person with virtual video sessions allowed for flexibility in scheduling and reduced the burden of traveling to sessions, which was significant for some participants with high symptom burden.

#### 3.4.3 Benefits of Completing the Program

Three major subthemes captured participants' perspectives after receiving the physiotherapy intervention: (1) confidence, (2) self‐management strategies, and (3) mental health benefits.

Many participants found the intervention endowed them with confidence and skills to help manage their symptoms. These skills often included pacing strategies and breathing techniques. Post‐intervention, participants found that these management strategies continued to help them improve their everyday functioning, which subsequently also led to increased confidence.

Many participants also found that following completion, they perceived an overall benefit to their mental health. This was potentially related to both improvement in their physical symptoms, as well as their newfound confidence and control over how they manage and cope with their symptoms. This theme was a stark contrast to perspectives before the intervention, where the predominant subthemes among participants were frustration and hopelessness.

## 4 Discussion

This study demonstrates that implementing an individualized respiratory physiotherapy program for persons with PASC and persistent dyspnea was feasible. Furthermore, significant improvements were observed in secondary endpoints, namely health‐related quality of life and daily functioning. In support of our findings are numerous small trials that involved inspiratory muscle and exercise training, leading to significant improvements in breathlessness following COVID‐19 [15, 32]. The results from the RECOVE trial found more significant improvements in muscle strength and PROMs within the group who underwent exercise training plus inspiratory muscle training compared to those who had self‐management recommendations [14]. Two recently published systematic reviews and meta‐analyses also concluded that pulmonary rehabilitation improves dyspnea, quality of life, physical function, and depressive symptoms in individuals with PASC [33, 34].

While this study found statistically significant improvements in health‐related quality of life measures, the clinical relevance is difficult to discern in the absence of validated MCIDs among those with PASC. Del Corral et al. sought to determine the MCID for the EQ‐5D index and EQ‐VAS among individuals with PASC and found that an improvement of at least 7.5 in EQ‐VAS had acceptable discriminative ability for individuals who had improvements following a respiratory muscle training program [35]. By this measure, 8 of our 13 participants exceeded this MCID in their EQ‐VAS; however, it is important to note that this MCID has not been externally validated to date. Among our study population, there was a moderate effect size observed in the differences in EQ‐5D index, EQ‐VAS, and PCFS.

There were excellent retention and completion rates during this study, and the qualitative analysis was invaluable for determining contributing factors as well as affirming the clinical benefit that coincided with statistically significant improvements in PROMs. We noted that several participants valued the flexibility in scheduling their sessions as well as the hybrid delivery of in‐person and virtual sessions. While all participants took more than 8 weeks to complete the program, this allowed more time to practice home exercises in between sessions, and the flexible scheduling facilitated completion of all sessions. Although telerehabilitation offers convenience, some participants in our study highlighted that in‐person sessions were more useful than virtual sessions. Many participants placed importance on having a therapist face‐to‐face to perform hands‐on assessments and demonstrations. A systematic review done by Martinez‐Pozas and colleagues found that both delivery modes improved outcomes; however, the in‐person format led to more significant improvement in the physical domain of quality of life compared to the telerehabilitation format [30]. A subsequent review and network meta‐analysis found that there were greater improvements in physical function and physical and mental quality of life with the in‐person format compared to telerehabilitation [36]. The mode of delivery may need to be individualized, with an option for hybrid models if possible.

Before receiving the intervention, participants experienced significant frustration and hopelessness related to diagnostic uncertainty and difficulty with managing symptoms. After the intervention, participants gained confidence related to the physical symptom management strategies they acquired from physiotherapy, which in turn led to mental health benefits. The impact described by participants is corroborated by the significant improvements in numerous outcome measures, including the PCFS and self‐efficacy scores for managing symptoms in various settings (the latter demonstrating a large effect size in all domains).

Our study is unique in that the cohort consisted only of those without pulmonary impairment detected on conventional testing. There are several potential contributors to unexplained dyspnea and exercise capacity in this population. A common mechanism is BPD following COVID‐19, which can have a prevalence as high as 21%–30% among those with PASC [37, 38]. As such, it is pivotal for physiotherapy programs to include dedicated breathing retraining exercises. This population can also suffer from autonomic dysfunction and PEM, which our program took into consideration when planning rehabilitation strategies. In addition, frailty because of COVID‐19 and/or hospitalization can compound symptoms. The terms “tailored” and “individualized” are loosely defined as they apply to studies of physiotherapy programs, as many are referring to the tailoring of pacing and intensity. However, the program within this study individualized the components of the intervention as well as the intensity and schedule, as it is unlikely to find a “one‐size‐fits‐all” solution for those with PASC.

There are several limitations to note in this small pilot study. The primary goal was to assess feasibility, and due to the lack of a control group to account for confounders, data showing improvements must be interpreted with caution. The impact of time alone cannot be ruled out; however, based on the long interim between initial infection and enrollment for most participants (ranging from 14 to 57 months), it is reasonable to suggest that benefits were derived from the relatively brief physiotherapy program. Additionally, the sample size was small, limiting the ability to detect more subtle differences within our study population. Larger studies are needed to determine if there is an impact on outcomes like 6MWD. Selection bias is also a possibility as the population in the study was identified from specialty clinics and may limit the generalizability to those receiving care in primary care settings. It is also important to note that confirmatory testing for COVID‐19 may no longer be as easily accessed as it was during the pandemic, and thus, our study's inclusion criteria of requiring a prior positive PCR test could exclude a relevant patient population. Nonresponse bias is also possible, given that some participants did not undergo interviews for the qualitative analysis, potentially skewing these results to appear more favorable towards the intervention. Finally, the sustainability of these improvements is unclear. Future studies should aim to incorporate longer‐term follow‐up to determine the duration of any impact.

The results of this pilot feasibility study suggest that more rigorous and powered studies are warranted to determine whether this novel approach to managing PASC could be beneficial and scalable to larger populations. This intervention was able to be implemented in an outpatient setting with minimal resources and was found to be safe and beneficial for participants. Based on our qualitative results, the authors support a personalized approach to dyspnea management, a blend of in‐person and virtual sessions, and the use of activity trackers to assist patients with pacing and exercises when designing future physiotherapy programs for individuals with PASC suffering from respiratory symptoms.

### 4.1 Implications for Physiotherapy Practice

This study demonstrates that an individualized physiotherapy program for persons with PASC is feasible, beneficial for symptom burden, and improves health‐related quality of life and daily functioning. The physiotherapy intervention was able to be implemented with minimal resources in an outpatient setting as a blend of in‐person and virtual sessions, which could be scalable to a larger population. As COVID‐19 continues to show seasonal resurgence, there will likely be a continuous population of individuals afflicted with PASC. Given that there are currently no approved treatments for PASC, implementing individualized physiotherapy programs that focus on education/self‐management strategies, breathing retraining, and return‐to‐activity exercise programming would likely be beneficial for this population.

## Author Contributions

**Marcus Lo:** investigation, writing – original draft, writing – review and editing, software, formal analysis. **Lauren Eiriksson:** investigation, writing – review and editing, data curation. **Simone Hunter:** data curation, writing – review and editing. **Rosie Twomey:** writing – review and editing, data curation. **Kate Skolnik:** writing – review and editing, conceptualization, methodology. **Joel Chen:** conceptualization, writing – review and editing, methodology. **Elnaz Ehteshami Afshar:** conceptualization, writing – review and editing, methodology. **Jason Weatherald:** conceptualization, writing – review and editing, methodology. **Rachel K. Lim:** conceptualization, investigation, funding acquisition, writing – original draft, writing – review and editing, methodology, validation, visualization, project administration, supervision, resources, software. All authors have read and approved the final version of the manuscript.

## Ethics Statement

This study protocol was reviewed and approved by the Conjoint Health Research Ethics Board at the University of Calgary, approval number REB21‐0767.

## Consent

Written informed consent was obtained from participants to participate in the study.

## Conflicts of Interest

The authors declare no conflicts of interest.

## Transparency Statement

The lead author, Rachel K. Lim, affirms that this manuscript is an honest, accurate, and transparent account of the study being reported; that no important aspects of the study have been omitted; and that any discrepancies from the study as planned (and, if relevant, registered) have been explained.

## Supporting information

POETIC‐Qualitative Interview Draft.

## Data Availability

The data that supports the findings of this study are available in the supplementary material of this article. R.K.L. had full access to all of the data in this study and takes complete responsibility for the integrity of the data and the accuracy of the data analysis.
