# Supplementary material for: Individually Tailored Physiotherapy in Persons With Respiratory Symptoms Related to Post‐Acute Sequelae of COVID‐19: A Feasibility Study With Mixed Methods
Source: Health Sci Rep. 2025 Oct 21;8(10):e71367. doi: 10.1002/hsr2.71367 (PMC12541134; doi:10.1002/hsr2.71367)
Supplement: Supplementary file 1 — POETIC‐Qualitative Interview Draft. [file HSR2-8-e71367-s001.docx]

**A pilot study of a PhysiOthErapy-based Tailored Intervention for long Covid (POETIC)**

**POETIC: Semi-Structured Interview Script**

Thank you for your involvement in the POETIC program. We want to find out what you liked and didn’t like about this program so that we can determine what changes we might want to make. We would like to make this program available to other people who have ongoing symptoms after COVID-19. We also want to make sure that people find it acceptable and beneficial. Today, we will talk about your experience with different aspects of the program. As a reminder, your anonymity and confidentially will be kept throughout the entire research process. I am going to record the audio of this discussion, and this will be stored in a confidential and secure manner. This interview should take about 15-45 minutes to complete. Should you need a break, we can stop at any time. You also have the ability to withdraw at any point from the interview. I have some questions prepared, but if you wish not to comment on something, that is perfectly fine. I was not involved in designing the intervention, there are no right or wrong answers, and all of your thoughts are important. Do you have any questions? (When ready, begin recording).

**Why did you decide to take part in the POETIC program?**

PROBES: Were there ways in which you thought being in this program would help you? If so, please explain. What were your expectations? Were your expectations met?

**In your opinion, what was the purpose of the program?**

PROBES: How was the program introduced to you? What was the aim of the program?

**What was your experience of taking part in this research?**

PROBES: What was your experience of recruitment; clinic assessments; the questionnaires.

**What was your experience of having sessions in-person/virtually?**

PROBES: What did you like/dislike about in-person? What did you like/dislike about virtual? Which did you prefer/favour and why? What were your barriers to meeting with the physiotherapist in-person/virtually? What made it easier for you to meet with the PT in-person/virtually?

**What were your most distressing symptoms before the program began?**

**Can you explain to me your confidence for managing your symptoms?**

PROBES: How did this change, if at all over the course of the intervention? In what ways are your symptoms better or worse? Please describe.

**Can you describe your capacity to do you usual daily activities?**

How did this change, if at all over the course of the intervention? How does this make you feel? In what ways is your overall function better or worse? What do you attribute to this change in function?

**How did the in *individual sessions* in the POETIC program affect you?**

PROBES: Thinking back to your individual sessions, how did you feel after each session? How did this differ from session to session? What about later that same day? For the rest of the week?

**Overall, how did taking part in the *POETIC program* impact you?**

PROBES: Why do you think this is? What do you attribute to these changed symptoms? Can you give me some examples?

**Can you share what aspects of the POETIC program you found most useful/beneficial?**

PROBES: Why? How did this aspect help you? Can you give me an example? Did you find any elements that were not useful? Can you please explain? What part did you like most?

**What did you find most challenging during the POETIC program?**

PROBES: Is this/are those thing(s) still hard for you now? What were the barriers you faced? What did you dislike?

**What changes did you need to make so that you could take part in the intervention?**

PROBES: Did you have to make changes to your schedule/mindset/expectations/daily activities?

**Can you tell me about how you integrated the recommendations from the physiotherapist within your day or week?**

PROBES: What helped you integrate recommendations during your day/week? What made it harder for you to use what you had learnt/practiced?

**Tell me about your experience monitoring your (1) heart rate (2) symptoms (3) activity during the program?** (Ask each separately)

PROBES: What did you like/dislike about this?

**Can you share what you thought about the number of sessions per week (typically 1 per week)?**

PROBES: Did you like/dislike this? Why? How could we improve this? If we were to change the number of sessions per week, what would be best for you?

**Can you share what you thought about the duration of individual sessions (typically 60 min)?**

PROBES: Did you like/dislike this? Why? How could we improve this? If we were to change the duration of sessions, what would be best for you?

**Can you share what you thought about how long the program lasted (typically 8 weeks)**

PROBES: Did you like/dislike this? Why? How could we improve this? If we were to change the length of the program, what would be best for you?

**Can you please tell me about your sessions with the physiotherapist?**

PROBES: How did they make you feel? What did they do that you like/don't like? How could we improve this?

**What did you learn about your symptoms and managing them during the intervention?**

PROBES: How are you currently using what you learned? Can you give me an example? What strategies are you not using? Why?

**What do you suggest we change to improve the *POETIC program*?**

PROBEs: Why would you change that? How would it make the program better? Anything else that you would add/remove?

**Is there anything else you'd like to tell me about the POETIC program or taking part in this research?**
